# Psychological wellbeing as a buffer against burnout and anxiety in academic achievement situations among physical education students

**DOI:** 10.3389/fpsyg.2025.1562562

**Published:** 2025-06-02

**Authors:** Jianye Li, Yuebo Li, Keqiang Li, Mariusz Lipowski, Zhan Shang, Dominika Wilczyńska

**Affiliations:** ^1^Faculty of Physical Education, Gdansk University of Physical Education and Sport, Gdańsk, Poland; ^2^Faculty of Education, Taiyuan Normal University, Jinzhong, China; ^3^Faculty of Sports Science, Wenzhou Medical University, Wenzhou, China; ^4^Faculty of Social and Humanities, WSB Merito University in Gdansk, Gdańsk, Poland; ^5^Department of Physical Education, Tianjin Chengjian University, Tianjin, China

**Keywords:** burnout, academic anxiety, wellbeing, physical education, mediation effect

## Abstract

**Introduction:**

This study examined the complex relationship between burnout, wellbeing, and academic achievement anxiety in physical education students. The focus was on how wellbeing moderates burnout and academic achievement anxiety.

**Methods:**

This study adopted a cross-sectional quantitative survey (n = 523) and used the Maslach burnout inventory–student survey (MBI-SS), Psychological wellbeing scale (PWBS), and Academic achievement anxiety test (AAT) to verify the mediation model of wellbeing among physical education students to identify the impact of wellbeing on burnout.

**Results:**

Using a range of psychological scales, we found significant positive correlations between self-acceptance and positive psychological dimensions such as Purpose in Life and Environmental Mastery. Facilitating anxiety was found to enhance self-acceptance and professional efficacy, while debilitating anxiety strongly correlated with burnout symptoms, including cynicism and exhaustion. Personal growth and autonomy were positively linked to psychological resilience and wellbeing. The study also highlighted the mediating role of psychological wellbeing in reducing the impact of burnout on academic anxiety.

**Discussion:**

These findings suggest that moderate anxiety can be motivating, but high levels of debilitating anxiety pose a significant risk for burnout. Interventions aimed at fostering emotional resilience, self-acceptance, and personal growth may help mitigate the effects of anxiety and burnout. The study’s limitations include its focus on a specific student population and cross-sectional design, which restricts generalizability and causal inferences. Future research should explore these relationships over time and in broader student populations, incorporating contextual factors and testing intervention effectiveness.

## 1 Introduction

Burnout is a pervasive issue that affects individuals across various professions and stages of life, including students in higher education. Among university students, particularly those majoring in physical education, the demands of academic and athletic performance often converge, creating unique stressors that can lead to burnout (Lee et al., 2017). Burnout is typically characterized by emotional exhaustion, cynicism, and a sense of reduced efficacy, as measured by the Maslach Burnout Inventory-Student Survey (MBI-SS) (Portoghese et al., 2018). These dimensions collectively impact students’ motivation, mental health, and academic performance, making burnout a critical area of research in educational and sports psychology (Salanova et al., 2010). Research by Schaufeli emphasizes that burnout not only undermines academic engagement but also correlates with long-term negative outcomes, including decreased life satisfaction and increased dropout rates (Schaufeli et al., 2009).

Academic achievement anxiety (AAT) is another critical concern for university students, as it directly influences their academic performance and overall educational experience. AAT is defined as a state of heightened worry and fear related to academic evaluations and performance outcomes. Excessive anxiety can impair cognitive functioning, reduce learning efficiency, and lead to avoidance behaviors (Robinson et al., 2013). Zeidner highlights that academic anxiety, while occasionally serving as a motivator, often results in significant psychological distress when experienced at high levels (Zeidner, 2014). In students majoring in physical education, this anxiety is often exacerbated by the dual pressures of academic and athletic success. Previous studies suggest that burnout and academic anxiety are interrelated, with higher levels of burnout often correlating with increased anxiety (Pokhrel et al., 2020). Despite the recognized relationship between burnout and anxiety, the mechanisms underlying this association remain underexplored, particularly in the context of physical education students.

Although burnout and academic anxiety have been studied independently in the context of physical education, few studies have systematically examined their interrelationships from a unified theoretical perspective or in the context of student mental health. Prior research has emphasized the psychological vulnerability of PE students. For example, Legey et al. (2017) showed that physically active students often experience symptoms of mood disorders and anxiety, which can hurt their quality of life. Similarly, Murphy et al. (2020) linked physical activity to improved mental health and decreased anxiety, depending on environmental and personal factors. In addition, Cox et al. (2011) and Yli-Piipari et al. (2009) also noted an association between physical activity and improvements in mental health and increased anxiety. However, these studies failed to explain the mechanisms by which chronic stressors such as burnout lead to persistent academic anxiety. Jaakkola et al. (2019) and Rogowska et al. (2020) also showed that achievement motivation and emotional coping affect anxiety and wellbeing, but neither explored the mediating role of mental health in this process.

To address this gap in theoretical and empirical research, the present study adopted Fredrickson’s (Fredrickson, 2003) Expansion and Construction Theory of Positive Emotions as a research framework. The theory suggests that positive emotions such as joy, hope, and fulfilment broaden an individual’s ability to think-act and contribute to the construction of long-term psychological resources. In the model, mental health is not only a mental health outcome but also a protective factor that mitigates the negative effects of stress and enhances emotional resilience. Applying this theory to academic settings, it is reasonable to expect that higher levels of mental health can reduce the emotional stress associated with burnout and alleviate academic achievement anxiety.

As a consequence, mental health has been identified as a potential mediator of the relationship between burnout and academic achievement anxiety. Mental health is rooted in the theoretical framework of positive psychology, which encompasses multiple dimensions such as self-acceptance, positive relationships with others, autonomy, environmental mastery, life goals, and personal growth, and is measured by the Psychological Wellbeing Scale (PWBS). Ryff’s model of psychological wellbeing (Ryff, 1995) highlights the importance of these dimensions in fostering resilience and mitigating the adverse effects of stressors like burnout. Enhanced psychological wellbeing may serve as a protective factor, buffering students from the negative emotional and cognitive impacts of burnout, thereby reducing academic achievement anxiety. Recent research supports the role of psychological wellbeing in promoting mental health and reducing vulnerability to stress (Charles, 2010).

The interrelation among these constructs—burnout, academic achievement anxiety, and psychological wellbeing—are critical for understanding the broader psychological and academic experiences of physical education students. Burnout, which drains emotional and cognitive resources, often leaves students vulnerable to heightened levels of anxiety (Dunn et al., 2008). Conversely, psychological wellbeing can offer a counterbalancing effect, promoting adaptive coping strategies, emotional regulation, and a sense of mastery. Similarly, academic achievement anxiety, while detrimental in excessive amounts, can also serve as a motivating factor when effectively managed (McCombs, 2001). Understanding how psychological wellbeing mediates the relationship between burnout and anxiety can provide deeper insights into these dynamics and offer pathways for targeted interventions.

Recent studies have further clarified the links between burnout, anxiety, and psychological wellbeing in academic contexts. For example, the Job Demands-Resources has been applied beyond workplaces to explain how excessive academic demands and limited support lead to student burnout and reduced wellbeing (Zhou et al., 2022). In university settings, psychological wellbeing and self-efficacy have been shown to enhance academic engagement, while high anxiety undermines performance (Lizarte Simón et al., 2024). These findings support the present study’s focus on wellbeing as a mediator between burnout and academic achievement anxiety.

The primary objective of this study is to explore the psychological mechanisms underlying the relationship between academic burnout and academic achievement anxiety among university students majoring in physical education. Specifically, the study aims to examine the direct relationships between academic burnout, psychological wellbeing, and academic achievement anxiety. Investigate whether psychological wellbeing mediates the relationship between academic burnout and academic achievement anxiety. And analyze whether there are significant differences in burnout, psychological wellbeing, and academic achievement anxiety across gender and education level.

Based on existing literature and the theoretical framework of Fredrickson’s broaden-and-build theory of positive emotions, the study proposes the following hypotheses:

H1: Academic burnout is positively associated with academic achievement anxiety.

H2: Academic burnout is negatively associated with psychological wellbeing.

H3: Psychological wellbeing is negatively associated with academic achievement anxiety.

H4: Psychological wellbeing mediates the relationship between academic burnout and academic achievement anxiety.

H5: There are significant differences in burnout, psychological wellbeing, and academic achievement anxiety across gender and education level.

By elucidating the mediating role of mental health through the lens of expanding and constructing positive emotion theory, the findings of this study have important implications for educators, counsellors, and policy makers. Can play a key role in creating more supportive and effective learning environments that ultimately promote students’ academic and psychological success.

## 2 Methodology

### 2.1 Procedure

Participants were recruited on a voluntary basis, and the questionnaire was administered by a faculty member on the research team to students at their university via an online link. A snowball sampling technique was used to encourage participants to invite all students who met the criteria. The research team followed the Guidelines for Self-Report Measures of Cross-Cultural Adaptation to ensure linguistic and cultural validity of the scale (Beaton et al., 2000). The questionnaire was translated into Chinese and its reliability and validity were confirmed using KMO and Bartlett tests. Sample size efficacy analyses were conducted using G*power (version 3.1). A minimum of 400 samples were required to achieve a medium effect size (*d* = 0.5), 80% efficacy and a significance level of 0.05. Ultimately 523 students participated in the study.

The survey consisted of basic demographic information about the participants and responses to three psychometric scales: the MBI-SS, PWBS, and AAT. The estimated time to complete the questionnaire was approximately 15 min.

### 2.2 Participants

The study was a descriptive cross-sectional study. The whole research involved a total of 523 university students majoring in physical education (271 males and 252 females). The average age of males was 20.178 ± 6.627 and that of females 19.351 ± 1.463. The participants in this study were all physical education majors from Tianjin Chengjian University and Taiyuan Normal University. The inclusion criteria for participants were current undergraduate or master’s degree students with a major in physical education. Participants who met the inclusion criteria received informed consent and an online link to the questionnaire.

### 2.3 Instruments

Three validated psychological scales, MBI-SS, PWBS, AAT, were used in this study. The study team adopted to the Guidelines for Cross-Cultural Adaptation of Self-Report Measures to ensure the linguistic and cultural validity of the scales. The content of the scales was translated into Chinese. And after full discussion by the study team to ensure the accuracy of the translated content.

#### 2.3.1 Maslach burnout inventory–student survey (MBI-SS)

First developed by Maslach and Jackson (1981), the Maslach Burnout Inventory (MBI) was initially used to measure burnout in occupational settings. Over time, the instrument was revised to suit different populations. The student-specific version, MBI-SS, was developed by Schaufeli et al. (2002) to assess burnout among university students. It comprises 15 items across three dimensions: Emotional Exhaustion (e.g., “I feel emotionally drained by my studies.”); Cynicism (e.g., “I have become less enthusiastic about my studies.”); Academic Efficacy (e.g., “I believe I am making effective contributions to the classes I attend.”). Items are rated on a 7-point Likert scale ranging from 0 (never) to 6 (always). Higher scores indicate higher levels of burnout. The internal consistency (Cronbach’s alpha) for the sample in this study was 0.916, Omega coefficient was 0.892.

#### 2.3.2 Psychological wellbeing scale (PWBS)

The PWBS was developed by Ryff and Keyes (1995) to assess multiple dimensions of psychological wellbeing. The short version used in this study includes 18 items covering six dimensions: Autonomy (e.g., “I have confidence in my opinions, even if they are contrary to the general consensus.”); Personal Growth (e.g., “I think it is important to have new experiences that challenge how you think about yourself and the world.”); Purpose in Life (e.g., “Some people wander aimlessly through life, but I am not one of them.”); Positive Relations (e.g., “People would describe me as a giving person, willing to share my time with others.”) Environmental Mastery (e.g., “In general, I feel I am in charge of the situation in which I live.”); Self-Acceptance (e.g., “I like most aspects of my personality.”). Participants responded on a 6-point Likert scale (1 = strongly disagree, 6 = strongly agree). Higher scores reflect higher levels of psychological wellbeing. The internal consistency (Cronbach’s alpha) in this study was 0.913, Omega coefficient was 0.823.

#### 2.3.3 Academic achievement anxiety test (AAT)

The Academic Achievement Anxiety Test (AAT), originally developed by Alpert and Haber (1960), is used to assess anxiety related to academic performance. It contains 19 items divided into two subscales: Facilitating Anxiety (e.g., “When I think about an upcoming exam, I feel more motivated to study.”) Debilitating Anxiety (e.g., “Even when I study hard for a test, I feel I will do poorly.”) Each item is rated on a 5-point Likert scale (1 = never, 5 = always). Higher total scores indicate higher levels of academic achievement anxiety. The internal consistency (Cronbach’s alpha) for this sample was 0.801, Omega coefficient was 0.789.

### 2.4 Data analysis

The data from the questionnaire were analyzed and validated using SPSS 26.0 (IBM, Armonk, New York, NY, USA) and AMOS 26.0 (IBM, Armonk, New York, NY, USA). Data visualization plots were generated in JupyterLab using Matplotlib^1^. The analysis was conducted in the following stages:

Stage 1: Data from the three scales were tested for normal distribution. The mean, standard deviation, skewness and kurtosis of the data were included to determine the subsequent statistical methods to be used. None of the three scales were tested to be normally distributed.

Stage 2: The correlations between the three scales were analyzed using Spearman’s correlation coefficient as the data did not conform to a normal distribution. Correlations were also analyzed between the dimensions of the scales.

Stage 3: A structural equation model was developed using AMOS 26.0, with burnout (MBI-SS) as the independent variable, psychological wellbeing (PWBS) as the mediating variable, and academic achievement anxiety (AAT) as the dependent variable. Before model estimation, key statistical assumptions for SEM were checked: Multivariate normality: Violated, as assessed in Stage 1. Multicollinearity: Variance inflation factors (VIFs) were below 2.0 for all latent indicators, indicating no multicollinearity issues. Error independence: Residual plots showed no discernible patterns, and the Durbin-Watson statistic (1.78) suggested acceptable error independence. Given the violation of multivariate normality, the maximum likelihood estimation with bootstrapping (5,000 samples) was used to obtain robust standard errors and confidence intervals. Model fit indices indicated acceptable fit: χ^2^/df = 1.432, CFI = 0.920, IFI = 0.910, RMSEA = 0.025. The model was tested to be a partial mediation effect.

Stage 4: Since the sample did not conform to normal distribution, the differences in scale scores were analyzed using non-parametric tests on two demographic characteristics, namely educational qualifications and gender. The test found significant differences in the MBI-SS scale by educational attainment. No significant differences were found by gender.

## 3 Results

### 3.1 Descriptive analysis

Table 1 shows the characteristics of the sample and the mean, kurtosis, skewness of each scale. There were 271 males with a mean age of 20.178 ± 6.627. The number of Bachelor was 109 and the number of master was 210. For females 252, the mean age was 19.351 ± 1.463. The number of Bachelor was 210 and the number of master was 111. The mean score of MBI-SS was 3.142 ± 1.138 for males and 3.106 ± 0.957 for females. The PWBS score was 4.484 ± 1.041 for males and 4.303 ± 0.757 for females. The AAT score was 4.484 ± 1.041 for males and 4.303 ± 0.757 for females. The AAT score was 4.484 ± 1.041 for females and 4.303 ± 0.757 for females. 0.757. The mean score of AAT was 2.901 ± 0.511 for males and 2.940 ± 0.381 for females. The kurtosis and skewness of all the variables showed that the samples did not conform to normal distribution.

**Table 1:** Characteristics of survey respondent.

Characteristic	Sample statistics	Gender	
		Male	Female
Age(years)	*n*	271	252
	Mean ± SD	20.178 ± 6.627	19.351 ± 1.463
	95% CI(*LL*)	19.387	19.170
	95% CI(*UL*)	20.968	19.532
	K	194.332	10.414
	S	13.180	2.032
Degree	Bachelor (*n*)	109	111
	Master (*n*)	210	93
MBI-SS	*n*	271	252
	Mean±SD	3.142 ± 1.138	3.106 ± 0.957
	95% CI(*LL*)	3.006	2.987
	95% CI(*UL*)	3.277	3.224
	K	0.519	−0.155
	S	−0.292	−0.285
PWBS	*n*	271	252
	Mean ± SD	4.484 ± 1.041	4.303 ± 0.757
	95% CI (*LL*)	4.360	4.209
	95% CI (*UL*)	4.608	4.396
	K	2.545	8.407
	S	0.461	−0.754
AAT	*n*	271	252
	Mean ± SD	2.901 ± 0.511	2.940 ± 0.381
	95% CI (*LL*)	2.841	2.893
	95% CI (*UL*)	2.962	2.987
	K	3.705	4.476
	S	−0.200	−0.489

SD = standard deviation; S = skewness; K = kurtosis.

### 3.2 Relations between PWBS, MBI-SS, AAT scores

**Figure 1:**
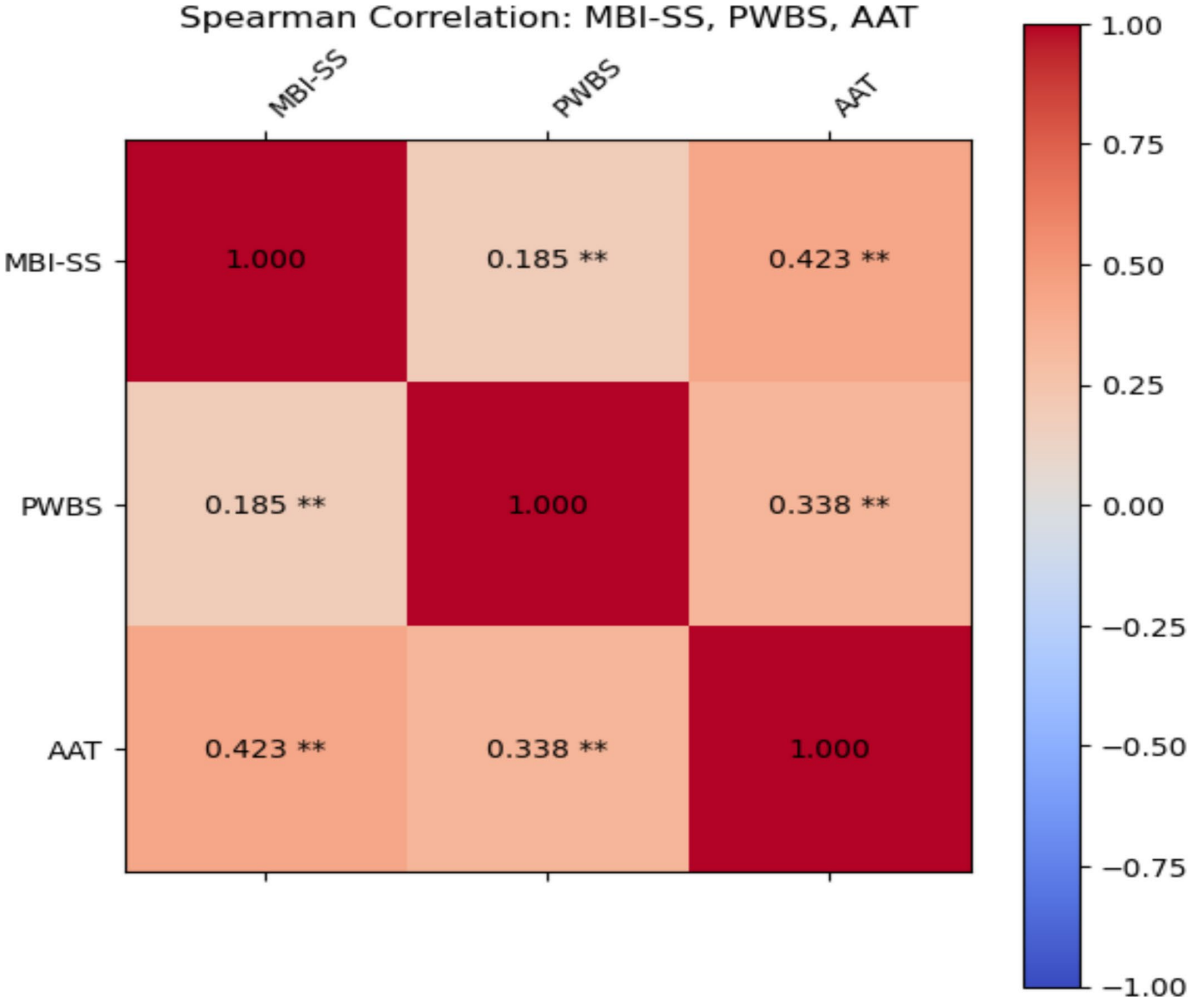
Spearman correlation with MBI-SS, PWBS and AAT.

Table 2 and Figure 1 shows MBI-SS the correlation coefficient with PWBS was 0.185: a weak positive correlation (*p* < 0.01). This indicates that there is a slight positive relationship between burnout and psychological wellbeing, possibly due to the fact that some students reassess their wellbeing when they perceive burnout, but the degree of correlation is low. The correlation coefficient with the AAT was 0.423: Moderately positive correlation (*p* < 0.01). It indicates that achievement anxiety has a strong positive association with burnout. The higher the achievement anxiety, the greater the likelihood of students feeling burnout, which is consistent with theoretical expectations.

**Table 2:** MBI-SS, PWBS and AAT Spearman correlation analysis.

Variables	MBI-SS	PWBS	AAT
MBI-SS	1		
PWBS	0.185**	1	
AAT	0.423**	0.338**	1

** *p* < 0.01.

The correlation coefficient between PWBS and AAT was 0.338: a moderate positive correlation (*p* < 0.01). It indicates that there is a significant positive relationship between achievement anxiety and psychological wellbeing. This may reflect the fact that with a certain level of anxiety, students are more likely to focus on academic tasks, thus enhancing the perception of wellbeing.

AAT showed positive correlation with both variables: Significant positive relationships were found with both MBI-SS and PWBS, especially with MBI-SS. This suggests that anxiety not only affects students’ burnout, but may also indirectly affect their psychological wellbeing.

There was a positive correlation between all the variables, which may reflect: students’ anxiety and burnout tendencies work together to some extent, while wellbeing feels its certain influence, but the strength of the correlation is low. Achievement anxiety played an important role in the model, both influencing burnout and significantly correlating with psychological wellbeing.

This Table 3 presents the results of Spearman correlation analysis among various psychological dimensions. Below is the main analysis and interpretation:

**Table 3:** Correlation coefficient analysis among the dimensions of MBI-SS, PWBS and AAT.

Variables	1	2	3	4	5	6	7	8	9	10	11
Self-acceptance (1)	1										
Purpose in life (2)	0.568**	1									
Positive relations with others (3)	0.404**	0.510**	1								
Personal growth (4)	0.474**	0.246**	0.285**	1							
Environmental mastery (5)	0.523**	0.391**	0.341**	0.559**	1						
Autonomy (6)	0.431**	0.294**	0.316**	0.678**	0.628**	1					
Debilitating anxiety (7)	0.077	0.287**	0.444**	0.067	0.099*	0.094*	1				
Facilitating anxiety (8)	0.506**	0.340**	0.230**	0.409**	0.459**	0.360**	0.079	1			
Professional efficacy (9)	0.429**	0.171**	0.061	0.552**	0.431**	0.438**	0.011	0.564**	1		
Cynicism (10)	−0.058	0.212**	0.318**	−0.107*	−0.004	−0.033	0.561**	−0.022	−0.169**	1	
Exhaustion (11)	−0.021	0.218**	0.322**	−0.075	0.055	0.027	0.579**	0.017	−0.108*	0.807**	1

*p < 0.05; **p < 0.01.

#### 3.2.1 Significant correlations among psychological dimensions

Self-Acceptance shows a significant positive correlation with other positive psychological dimensions, such as Purpose in Life and Environmental Mastery: Purpose in Life: (*r* = 0.568, *p* < 0.01). Environmental Mastery: (*r* = 0.523, *p* < 0.01). This indicates that higher levels of self-acceptance are associated with stronger perceptions of life purpose and environmental control.

Positive Relations with Others shows a weaker but significant correlation with Facilitating Anxiety: (*r* = 0.230, *p* < 0.01). This suggests that facilitating anxiety might help students build better social connections to some extent.

#### 3.2.2 Role of different types of anxiety

Facilitating Anxiety: Positively correlated with Self-Acceptance (*r* = 0.506, *p* < 0.01) and Professional Efficacy (*r* = 0.564, *p* < 0.01). This suggests that moderate levels of anxiety can enhance students’ confidence and sense of achievement.

Debilitating Anxiety: Strongly correlated with Cynicism and Exhaustion (*r* = 0.561, *p* < 0.01; *r* = 0.579, *p* < 0.01). This indicates that debilitating anxiety is a key predictor of burnout.

#### 3.2.3 Impact of burnout dimensions

Cynicism and Exhaustion: Strongly correlated with each other (*r* = 0.807, *p* < 0.01). Both are negatively correlated with Professional Efficacy (*r* = −0.169, *p* < 0.01; *r* = −0.108, *p* < 0.05). This shows that severe burnout significantly reduces students’ sense of professional efficacy.

#### 3.2.4 Personal growth and autonomy dimensions

Personal Growth and Autonomy: Show significant positive correlations with Environmental Mastery (*r* = 0.559, *p* < 0.01; *r* = 0.628, *p* < 0.01). This highlights the critical role of personal growth and autonomy in maintaining psychological stability.

### 3.3 Structural model analysis

**Figure 2:**
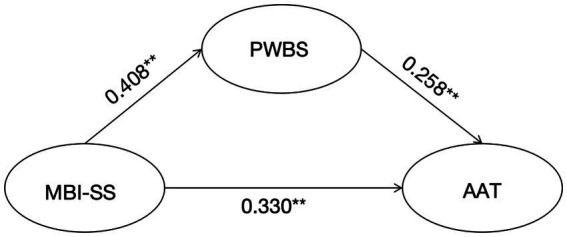
PWBS as a mediating variable in the mediation model between MBI-SS and AAT. Values represent the significant standardized regression coefficients. **p < 0.01.

Figure 2 shows that the model was constructed using MBI-SS as the independent variable, AAT as the dependent variable and PWBS as the mediator variable. Analysis of the model using AMOS 26.0 shows good model fit with x^2^/df value of 1.432 which is less than 2 indicating good fit between the problem items. The Root Mean Square Error of Approximation (RMSEA) value is 0.025, which is less than 0.05, indicating a good model fit. The Comparative Fit Index (CFI) value is 0.920 and the Incremental Fit Index (IFI) value is 0.910, both of which are more than 0.9, which further proves that the model is well fitted.

The Table 4 and Figure 2 demonstrates the results of a bootstrap analysis to evaluate the mediation effect of PWBS in the relationship between MBI-SS and AAT. Below is an interpretation of the findings:

1. Indirect Effect (a *b): Effect Size is 0.134, *p* < 0.01. Confidence Interval (CI) is [0.146, 0.297]. This significant indirect effect indicates that MBI-SS affects AAT indirectly through PWBS. The confidence interval does not include 0, confirming the mediation effect. The path (a*b) is thus considered statistically significant.
2. Path a: MBI-SS → PWBS (Burnout to Wellbeing): Effect Size is 0.408, *p* < 0.01. Confidence Interval is [0.342, 0.473]. This positive standardized effect suggests that as burnout (MBI-SS) increases, psychological wellbeing (PWBS) significantly decreases.
3. Path b: PWBS → AAT (Wellbeing to Anxiety): Effect Size is 0.330, *p* < 0.01.
4. Confidence Interval is [0.289, 0.370]. This shows that higher levels of psychological wellbeing are associated with reduced anxiety levels in the academic context.
5. Direct Effect (c’): MBI-SS → AAT (Burnout to Anxiety Without Mediation): Effect Size is 0.258, *p* < 0.01. Confidence Interval is [0.223, 0.294]. This significant positive direct effect indicates that burnout directly contributes to academic anxiety.
6. Total Effect (c): MBI-SS → AAT (Burnout to Anxiety, Including Mediation): Effect Size is 0.393, *p* < 0.01. Confidence Interval is [0.355, 0.431]. This total effect encompasses both the direct and indirect contributions of burnout to academic anxiety.

**Table 4:** Bootstrap analysis of the mediation effect size and significance test of PWBS in MBI-SS and AAT.

Path			**Standardized effect size**	95% CI		*p*	Verdict
				LL	UL		
MBI-SS= > PWBS= > AAT	a*b	Indirect effect	0.134**	0.146	0.297	0.000	Partial mediation
MBI-SS= > PWBS	a	X= > M	0.408**	0.342	0.473	0.000	
PWBS= > AAT	b	M= > Y	0.330**	0.289	0.370	0.000	
MBI-SS= > AAT	c’	Direct effect	0.258**	0.223	0.294	0.000	
MBI-SS= > AAT	c	Total effect	0.393**	0.355	0.431	0.000	

CI, confidence interval; LL, lower limit; UL, upper limit. **p < 0.01.

Partial Mediation: The indirect effect (a*b = 0.134) and direct effect (c’ = 0.258) are both significant, suggesting that PWBS partially mediates the relationship between MBI-SS and AAT. Psychological wellbeing acts as a buffer, reducing the impact of burnout on anxiety.

Importance of PWBS: The strong path coefficients (a = 0.408), (b = 0.330) highlight the pivotal role of wellbeing in mitigating the negative effects of burnout on academic anxiety.

Impact of Burnout: The significant direct effect (c’) underscores that even without mediation, burnout is a significant predictor of anxiety.

The results support a partial mediation model, where PWBS reduces the adverse effects of burnout on academic anxiety. Interventions aimed at improving psychological wellbeing can help mitigate the impact of burnout and anxiety in students.

### 3.4 Intergroup differences analysis

**Figure 3:**
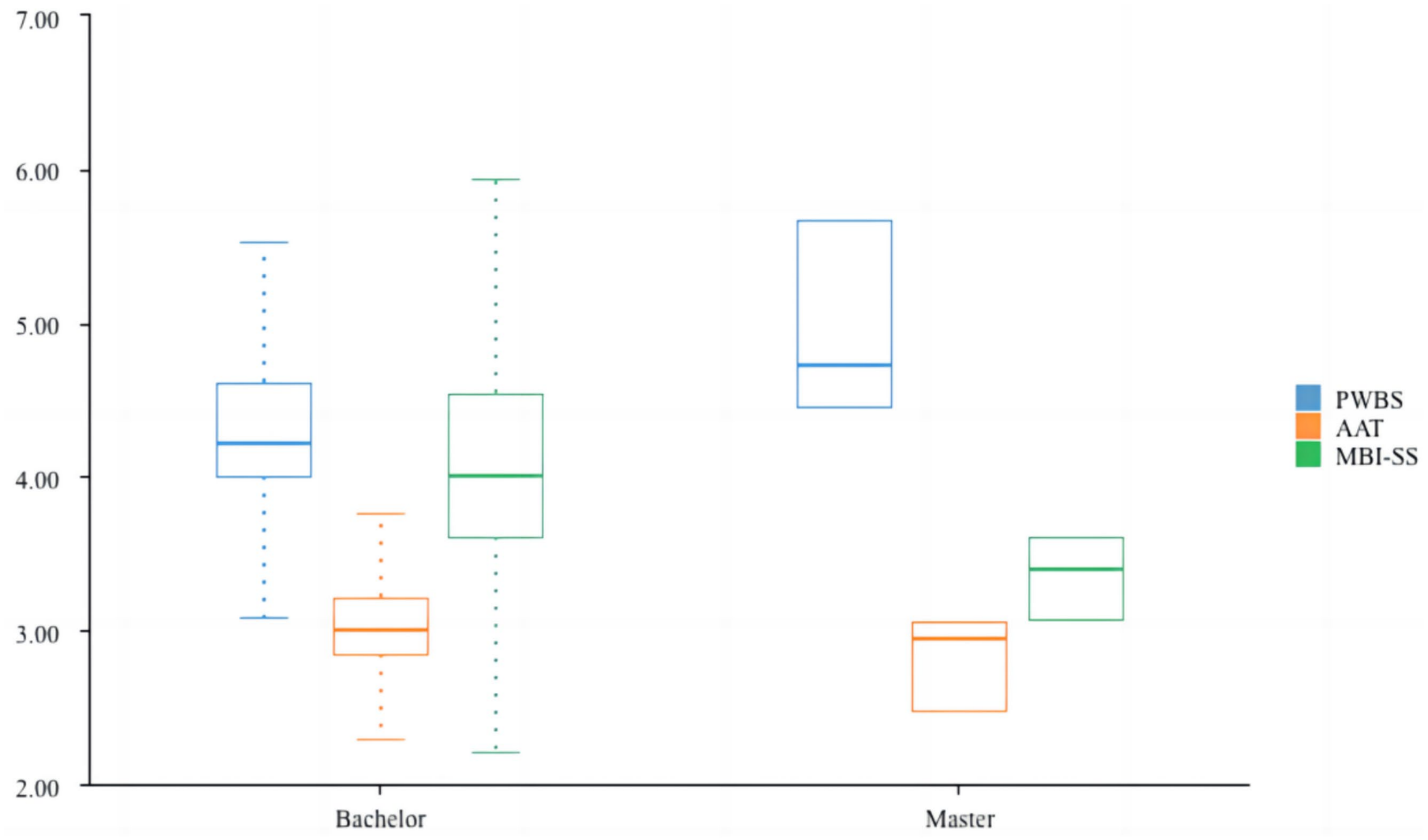
Non-parametric test of academic degree.

This Table 5 and Figure 3 presents the results of a Mann–Whitney *U* test, a non-parametric test used to compare differences between two independent groups (Bachelor’s vs. Master’s students) across three variables: PWBS (Psychological Wellbeing Scale), AAT (Achievement Anxiety Test), and MBI-SS (Maslach Burnout Inventory–Student Survey). There is a significant difference in burnout levels (MBI-SS) between Bachelor’s and Master’s students, with Bachelor’s students experiencing higher burnout. Interventions to reduce burnout may need to target Bachelor’s students specifically, as they appear to be more susceptible compared to Master’s students.

**Table 5:** Analysis of non-parametric tests of academic degree.

Variables	Degree		MannWhitney *U*	MannWhitney *z*	*p*
	Bachelor (*n* = 220)	Master (*n* = 330)			
PWBS	4.222	4.722	330.500	−1.732	0.083
AAT	3.000	2.947	559.500	−0.848	0.396
MBI-SS	4.000	3.400	251.500	−2.027	0.043*

* *p* < 0.05.

**Figure 4:**
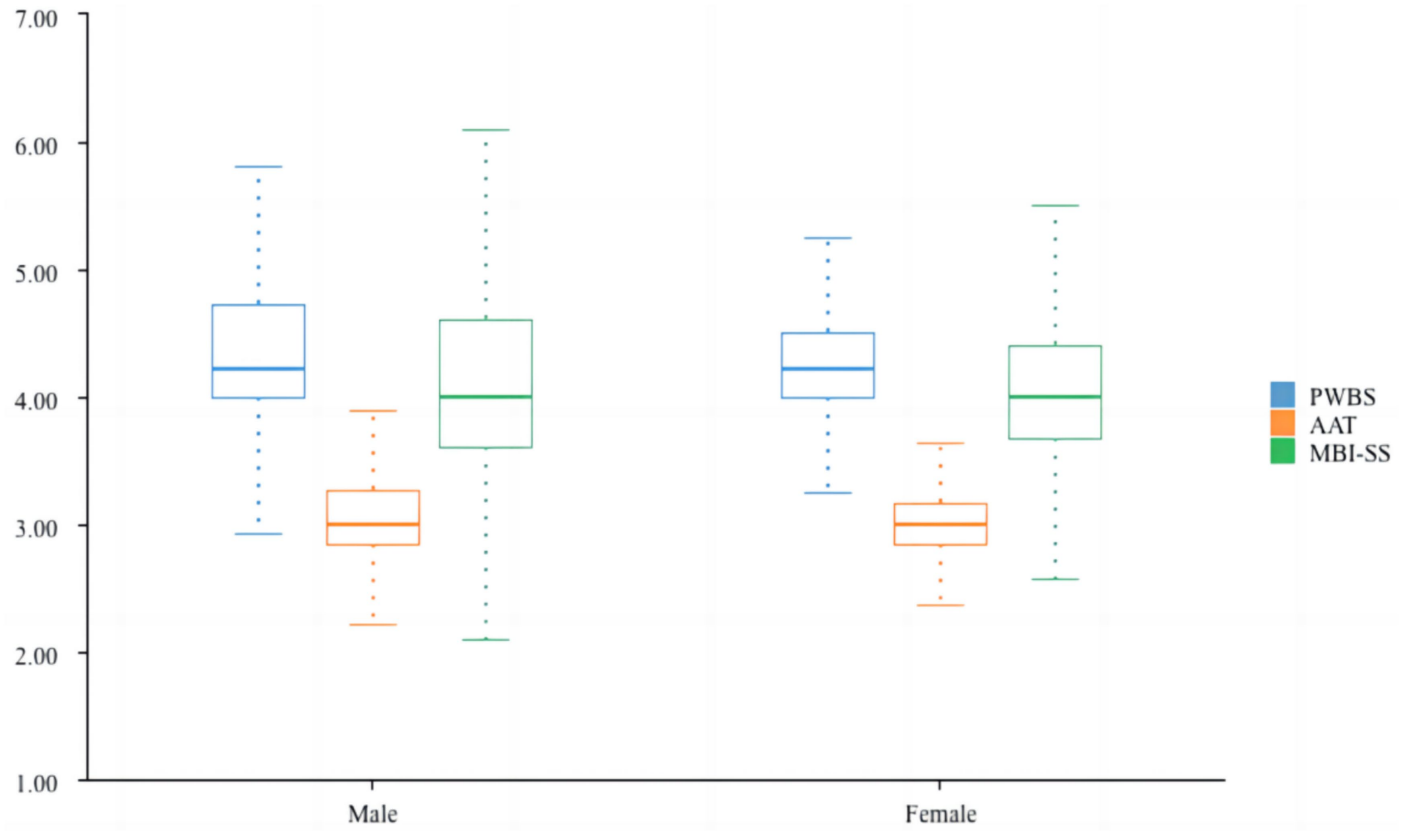
Non-parametric test of gender.

This Table 6 and Figure 4 shows the results of a Mann–Whitney *U* test, a non-parametric statistical method, used to compare the differences between males and females across three variables: PWBS (Psychological Wellbeing Scale), AAT (Achievement Anxiety Test), and MBI-SS (Maslach Burnout Inventory–Student Survey). No statistically significant differences were found between males and females for any of the three variables (PWBS, AAT, MBI-SS). Both genders exhibit similar patterns in wellbeing, anxiety, and burnout. Gender does not appear to be a significant factor influencing wellbeing, academic anxiety, or burnout in this sample. Interventions and support programs targeting these variables can likely be implemented without a specific gender focus.

**Table 6:** Analysis of non-parametric tests of gender.

Variables	Gender		MannWhitney *U*	MannWhitney *z*	*p*
	Male (*n* = 271)	Female (*n* = 252)			
PWBS	4.222(4.0,4.7)	4.222(4.0,4.5)	32735.000	−0.822	0.411
AAT	3.000(2.8,3.3)	3.000(2.8,3.2)	32550.500	−0.928	0.353
MBI-SS	4.000(3.6,4.6)	4.000(3.7,4.4)	33708.500	−0.254	0.800

## 4 Discussion

This study explored the relationship between burnout, Psychological wellbeing and Academic achievement anxiety among Physical Education (PE) students. Findings revealed significant associations between these psychological dimensions, with Psychological wellbeing playing a key role in moderating the impact of burnout on Academic achievement anxiety. Furthermore, differences in burnout levels between undergraduate and masters students highlight the need for targeted interventions. Below, we summarize the key findings for PE students.

### 4.1 Burnout and psychological wellbeing correlation analysis

The study revealed a weak positive correlation between burnout (MBI-SS) and Psychological wellbeing (PWBS). For physical education students, this suggests that whilst burnout may slightly affect their perception of wellbeing, other factors, such as their level of physical activity and peer interactions, may also impact on their wellbeing (Pierannunzio et al., 2022). Burnout and Academic achievement anxiety showed a stronger correlation, suggesting that PE students’ academic anxiety increased as burnout levels rose. This finding is consistent with previous research suggesting that PE students face unique academic pressures to juggle practical and theoretical courses, which may lead to higher levels of burnout and Academic achievement anxiety (Oliveira et al., 2021; Agortey, 2023).

The moderate correlation between Psychological wellbeing and Academic achievement anxiety suggests that PE students with better Psychological wellbeing status may experience lower levels of Academic achievement anxiety. This is particularly important in physical education, where the demands of both physical education and academic performance create additional stress. A sense of wellbeing can act as a buffer, allowing students to manage stress more effectively and focus on academic tasks (Riolli et al., 2012; Zee and Koomen, 2016).

This study further explored the relationship between Self-Acceptance, Academic achievement anxiety, burnout, and psychological growth on three scales of Physical Education students, revealing their Psychological wellbeing status. Self-Acceptance and Positive Psychological Dimensions: Self-Acceptance was positively correlated with Purpose in Life and Environmental Mastery, highlighting the role of Self-Acceptance in contributing to overall wellbeing. For physical education students, Self-Acceptance is critical to the academic and physical demands of the physical education major. It enhances life satisfaction and coping strategies and helps students cope with the stress of training and studying (Mahmoud et al., 2012; Chan and Huang, 2024).

Role of Facilitating Anxiety: Facilitating Anxiety is a motivator of achievement and is positively correlated with Self-Acceptance and Professional Efficacy This moderate anxiety can motivate physical education students and improve their focus, performance, and confidence in academic and athletic tasks ([Bibr ref26][Bibr ref26]). It can be a motivating factor rather than a hindering factor.

Effects of Debilitating Anxiety: Debilitating Anxiety is closely related to Cynicism and Exhaustion, which are major components of professional burnout. For physical education majors, high levels of anxiety can lead to disengagement and emotional exhaustion, increasing the risk of career burnout and decreasing Professional Efficacy (Lee et al., 2021; Rigg et al., 2013).

The occupational burnout dimension: Cynicism and Exhaustion are closely related, with higher levels of occupational burnout being associated with lower Professional Efficacy. In physical education, where students juggle physical education and academic responsibilities, professional burnout can seriously affect their confidence and performance (Mauzy et al., 2015; Smith and Leng, 2003; Ellison and Woods, 2019).

Personal Growth and Autonomy: personal growth and environmental mastery (*r* = 0.559, *r* = 0.628) were positively correlated, highlighting the importance of Personal Growth and Autonomy for psychological stability. These factors help physical education students manage academic and athletic stress and contribute to resilience and wellbeing (Pasno, 2024).

This study highlights the need for interventions that address both Facilitating Anxiety and Debilitating Anxiety. Moderate Anxiety can increase self-acceptance and productivity, whereas Debilitating Anxiety can lead to professional burnout. Interventions should focus on developing emotional resilience, self-acceptance, personal growth and autonomy to support the wellbeing of physical education students and prevent burnout (Trigueros et al., 2019; Gilar-Corbi et al., 2024). Future research should explore the long-term effects of these psychological factors, as well as effective physical activity strategies for managing the unique demands of the physical education curriculum, which can serve as an outlet for stress (Bailey et al., 2009). Encouraging PE students to balance their academic responsibilities with physical activity can help mitigate burnout and its impact on academic performance.

Recent research has emphasized the importance of integrating psychological constructs such as resilience and grit into the discussion of students’ mental health and academic performance, especially in physically and cognitively demanding disciplines like physical education. Trigueros and García-Mas (2025) argue that psychological wellbeing is deeply connected to self-determined motivation, perseverance, and the capacity to adapt to challenges. Their study highlights the concept of novelty—introducing new, engaging, and stimulating experiences in physical education—as a key factor in promoting students’ psychological health. These findings offer a valuable complement to the present study by suggesting that innovative and varied instructional strategies may serve as protective environmental factors that foster resilience and reduce emotional exhaustion. In this context, interventions aiming to prevent burnout and reduce academic achievement anxiety should not only focus on internal coping strategies but also consider external motivational factors, such as diverse and meaningful learning experiences. Supporting psychological wellbeing in physical education students therefore requires a holistic approach that incorporates personal strengths (e.g., grit and resilience) and supportive academic environments.

Additionally, creating a supportive environment that promotes Psychological wellbeing, including peer support groups and Psychological wellbeing services, can mitigate the negative effects of anxiety and burnout. Given the nature of physical education programs that require mental and physical endurance, holistic interventions that target the psychological and physical aspects of students’ lives can significantly improve their overall wellbeing (Lubans et al., 2012).

In conclusion, this study suggests that Psychological wellbeing plays an important role in moderating the relationship between burnout and academic anxiety in PE students. The findings highlight the need to develop tailored interventions for burnout, particularly for undergraduate students who are more vulnerable to burnout. By promoting Psychological wellbeing and providing stress management strategies, physical education programs can help students better cope with the unique demands of academics and athletic training, ultimately improving their academic performance and Psychological wellbeing. Future research should continue to explore the relationship between Psychological dimensions and specific challenges faced by physical education students. Complex relationships.

### 4.2 The mediating role of psychological wellbeing

Lead-in analyses confirmed that Psychological wellbeing partially mediated the relationship between burnout and Academic achievement anxiety among PE students. Burnout had a direct effect on Academic achievement anxiety was significant, emphasizing that burnout directly leads to Academic achievement anxiety, even when mediated. However, the mediating effect highlighted the role of Psychological wellbeing in reducing Academic achievement anxiety. This suggests that interventions aimed at improving wellbeing (e.g., positive thinking or stress reduction strategies) can help PE students cope with the academic pressures they face, both in terms of the physical demands of the course and the profession (Armour, 2010). The overall effect represents the combined effects of direct and indirect pathways from burnout to Academic achievement anxiety. The overall effect supports the idea that burnout significantly affects Academic achievement anxiety, with Psychological wellbeing being an important mediator. The magnitude of the overall effect suggests that although burnout has a significant direct effect on Academic achievement anxiety, its effect can be partially mitigated by improving students’ Psychological wellbeing. Burnout was associated with wellbeing and wellbeing was associated with Academic achievement anxiety. The strong path coefficients between burnout and wellbeing and wellbeing and academic achievement anxiety emphasize the importance of developing Psychological wellbeing as a protective factor. Given that physical education programs combine the dual nature of physical training and academic learning, enhancing wellbeing may reduce the detrimental effects of burnout on Academic achievement anxiety and promote physical and mental health (Cadime et al., 2016).

The findings support the need for interventions aimed at improving Psychological wellbeing as a strategy to reduce the adverse effects of burnout on Academic achievement anxiety. Given the significant indirect effects of Psychological wellbeing, interventions that focus on improving students’ wellbeing (e.g., positive thinking training, stress management programs, and promotion of a sense of purpose and achievement) can be effective in mitigating burnout and Academic achievement anxiety (Wei et al., 2021). Additionally, interventions that directly assist students in managing burnout through time management skills, social support, and promoting a balanced workload are critical to reducing academic anxiety (Chemagosi, 2024). In summary, the results of the bootstrap analyses suggest that Psychological wellbeing plays a key role in buffering the negative effects of burnout on Academic achievement anxiety, supporting the partial mediation model. This highlights the importance of fostering Psychological wellbeing as part of a comprehensive strategy to reduce students’ burnout and Academic achievement anxiety. Further research should explore other mediators, such as coping strategies or social support, and examine how these can be integrated into interventions to promote Psychological wellbeing and resilience in academic settings.

### 4.3 Differences between undergraduate and masters students

The results of the current study revealed a significant difference in burnout levels between undergraduate and masters students, with undergraduates reporting higher levels of burnout. This finding is consistent with previous research which suggests that undergraduate students enrolled in physically demanding programs such as sport tend to experience higher levels of stress when transitioning into higher education (Wrench et al., 2014). Undergraduate students may have difficulty adapting to rigorous academic requirements and practical training, which could explain their higher levels of burnout compared to master’s students who may have developed better coping mechanisms through prior experience.

On the other hand, master’s students may have acquired more resilience and coping strategies as a result of advanced courses and training, which helped them to better manage the combined stress of academic and physical performance (Wunsch et al., 2021; Rudnik et al., 2021). These differences emphasize the need for interventions tailored to each academic level, particularly for undergraduate PE students who are more prone to burnout.

The results suggest that interventions targeting burnout and Psychological wellbeing should be prioritized for PE students, particularly undergraduate students. As burnout has a direct impact on Academic achievement anxiety, it is important to provide support systems to reduce burnout through stress management techniques, time management workshops and opportunities.

### 4.4 Limitations of study

Despite its valuable findings, this study has several limitations. First, the sample was limited to physical education students, which restricts the generalizability of the results to other academic fields. Second, the cross-sectional design prevents causal conclusions; future longitudinal studies are needed to track changes over time. Third, the use of self-reported data may introduce biases such as social desirability or inaccurate self-assessment. Incorporating objective measures in future research could enhance validity. Addressing these limitations will help deepen understanding and improve targeted interventions for student wellbeing.

## 5 Conclusion

This study provides valuable insights into the Psychological wellbeing of Physical Education students, focusing on the complex relationship between Self-Acceptance, anxiety, burnout and personal growth. Findings highlight the importance of Self-Acceptance in fostering positive psychological outcomes, such as Purpose in Life Environmental Mastery, which is critical in addressing the academic and physical challenges faced by students in this field. Moderate levels of Anxiety, when considered Facilitating Anxiety, can serve as a motivational force that enhances Self-Acceptance and Professional Efficacy. However, high levels of Debilitating Anxiety are strongly associated with Burnout, manifested as Cynicism and Exhaustion, which can undermine students’ confidence and engagement in academic and sporting tasks. These findings emphasize the importance of managing Academic achievement anxiety levels to prevent burnout and maintain Psychological wellbeing. The study also highlights the key role of Personal Growth and Autonomy in promoting resilience and coping with stress. By developing these dimensions, PE students can better cope with professional stress and maintain Psychological wellbeing. Overall, the results suggest that interventions aimed at enhancing emotional resilience, Self-Acceptance, and Personal Growth can help alleviate Academic achievement anxiety and burnout in PE students. Future research should focus on exploring the long-term effects of these psychological factors and developing tailored customized strategies to support students with the unique challenges they face.

## Data Availability

The raw data supporting the conclusions of this article will be made available by the authors, without undue reservation.

## Footnotes

^1^ https://jupyter.org/try-jupyter/lab/
